# Increased Mitochondrial Activity in Anthrax-Induced Cell Death

**Published:** 2009-09-04

**Authors:** Chi Li

**Affiliations:** Molecular Targets Group, James Graham Brown Cancer Center, Department of Pharmacology and Toxicology, University of Louisville, KY 40202.

**Keywords:** anthrax, mitochondria, F_1_F_0_ ATPase, pyroptosis

## Abstract

Pathogenesis of anthrax lethal toxin (LT) is attributed to its ability to cause death of infected cells. New work has demonstrated that increase of mitochondrial F_1_F_0_ ATPase activity and subsequent depletion of cellular ATP level are critical early events during LT-induced cell death.

Toxins produced by the Gram-positive bacterium *Bacillus anthracis* are believed to be the primary virulence factors for three forms of anthrax: inhalational, cutaneous, and gastrointestinal.^1^ Anthrax toxins consist of three monomeric proteins: edema factor (EF), lethal factor (LF), and protective antigen (PA). EF is a Ca^2+^ and calmodulin-dependent adenylate cyclase while LF is a zinc-metalloprotease whose proteolytic activity is essential for its lethal effect. The third member, PA, is a receptor-binding and pore-forming protein. Anthrax edema toxin is composed of PA and EF, which induces an increase of cAMP, an important cellular second messenger, and subsequent tissue edema. On the other hand, PA and LF form anthrax lethal toxin (LT) that is responsible for cell death during systemic anthrax infection. By binding specific receptors on the plasma membrane (ANTXR1 or ANTXR2), PA mediates a Trojan Horse-like endocytosis of EF and LF. Anthrax toxins target many cell types in the immune system of the host to enable bacterial survival and proliferation. Through such mechanisms, anthrax toxins suppress macrophage function in the lung, the skin, and gut mucosa. Although effects on macrophages do not account for all of anthrax toxicity, compromising macrophage function is an essential step for the bacteria to evade the host immune system. Progress has been made on how anthrax LT causes macrophage cell death, which has been recently attributed to expression of a highly polymorphic gene, *Nalp1b*.^2^ The protein product, NALP1, recruits caspase-1 to the multimeric inflammasome assembled on the cytoplasmic membrane and promotes the activation of caspase-1.^3^,^4^ However, early events triggering LT-induced cell death are largely unknown. Emerging evidence indicates that cellular ATP pool depletion is important to cell death in LT-treated macrophages, although the link between LT and ATP depletion has been unclear. In this issue, Boldogh and colleagues provide compelling evidence that LF enhances F_1_F_0_ ATPase activity via directly interacting with β and γ subunits of the mitochondrial F_1_F_0_ ATPase complex. The resultant ATPase activation leads to ATP depletion, a pivotal early event in cell death induced by LT.^5^

Because the level of intracellular ATP plays an important role in rapid cytolysis (termed pyroptosis), including LT-induced cell death, Woodberry et al investigated the specific role of ATP depletion in cell death triggered by LT. First, they demonstrate that LT treatment causes ATP depletion and opening of the mitochondrial permeability transition pore (MPTP), followed by subsequent cell death in susceptible macrophages, but not in non-susceptible macrophages. The correlation between ATP depletion and MPTP opening led them to explore the possible direct interaction between LT and mitochondrial membrane protein complexes. Through a series of carefully executed experiments, they demonstrate that LF, but not PA, directly binds to subunits β and γ of F_1_F_0_ ATPase complex to enhance ATPase activity. Importantly, p^0^ cells, in which mitochondrial ATPase activity is compromised due to the depletion of mtDNA and subsequent deficiency in ATPase subunits F_0_6 and F_0_8, are more resistant to LF-triggered ATP depletion and cell death, providing evidence that inhibition of F_1_F_0_ ATPase activity may protect cells from LT-induced pyroptosis.

The investigations of Woodberry et al suggest a model in which cellular ATP depletion caused by anthrax LF-mediated enhancement of mitochondrial F_1_F_0_ ATPase activity is an important early event leading to LF-induced cell death (Fig. 1). This model is consistent with previous findings that ATP depletion plays a critical role in the life/death decision in various forms of cell death.^6^ This study has important implications as it may suggest pharmacologic approaches to protect cells from pyroptosis, perhaps by disrupting the interaction between F_1_F_0_ ATPase complex and LF or by decreasing the ATPase activity of F_1_F_0_. Probably the most interesting implication of this model is that ATP depletion leads to cellular K^+^ efflux, which is believed to be critical for assembly of NALP1 inflammasome and activation of downstream cell death signaling pathway.^7^ In contrast to a previous study suggesting that LT-associated efflux of K^+^ increases Na^+^/K^+^ pump activity and causes cellular ATP depletion,^8^ the authors provide evidence that Na^+^/K^+^ ATPase is an unlikely suspect because an inhibitor of Na^+^/K^+^ ATPase, ouabain, failed to prevent ATP depletion and subsequent death of LT-treated macrophages. Inasmuch as ATP depletion occurs in cells whose plasma membrane is still intact, they conclude that efflux of K^+^ via opening of K^+^ channels on the plasma membrane is the consequence, not the cause, of ATP depletion. However, it remains to be shown how ATP depletion stimulates K^+^ release in LF-infected macrophages.

The new work by the Boldogh group raises a number of important questions. For instance, LF is a metalloprotease whose physiological substrates include mitogen-activated protein kinase kinases (MAPKK) 1 and 2.^9^ Whether proteolytic cleavage of MAPPK is critical to the pathogenesis of anthrax is not known. Therefore, one critical question to be addressed is whether the proteolytic activity of LF is related to its ability to modulate F_1_F_0_ ATPase activity. *In vitro* far-western and immunoprecipitation experiments carried out by the authors provide convincing evidence that LF directly interacts with F_1_F_0_ ATPase. However, another unsolved issue in the proposed model is how LF penetrates the mitochondrial outer membrane and gets access to the F_1_F_0_ complex located on the mitochondrial inner membrane. It is intriguing to speculate that LF might utilize some existing protein translocation system on the mitochondrial outer membrane, such as the translocase of the outer membrane (TOM) complexes, to pass through the mitochondrial outer membrane.^10^

The work by Woodberry et al provides fundamental insights into the pathogenesis of anthrax in macrophages. Future studies will help to further elucidate the signaling pathways involved in LT induced-cell death and, perhaps, suggest new therapeutic strategies.

## Figures and Tables

**Figure 1:**
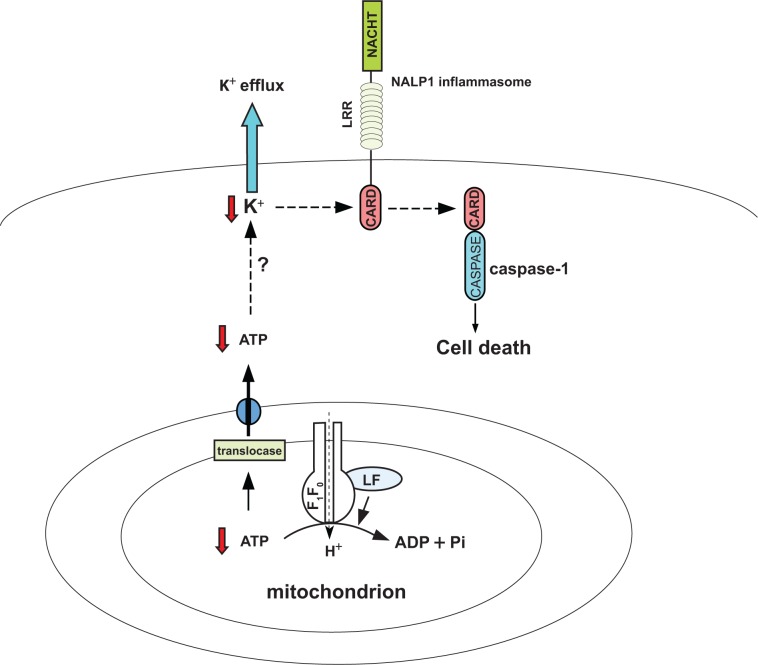
Cell death triggered by *B. anthracis* lethal factor. The model shows how lethal factor (LF) from *B. anthracis* induces cell death in susceptible macrophages. LF physically interacts with β and γ subunits of the mitochondrial F_1_F_0_ ATPase complex, leading to increased ATPase activity and subsequent depletion of intracellular ATP level. Through unknown mechanisms, ATP depletion results in cellular K^+^ efflux followed by formation of NALP1 inflammasome, activation of caspase-1, and ultimately cell death.
